# Abnormal Subcortical Brain Morphology in Patients with Knee Osteoarthritis: A Cross-sectional Study

**DOI:** 10.3389/fnagi.2016.00003

**Published:** 2016-01-19

**Authors:** Cui Ping Mao, Zhi Lan Bai, Xiao Na Zhang, Qiu Juan Zhang, Lei Zhang

**Affiliations:** ^1^Department of Medical Imaging, the Second Affiliated Hospital of Xi’an Jiaotong University College of Medicine, Xi’an, China

**Keywords:** knee osteoarthritis, subcortical structure, caudate nucleus, vertex analysis, FSL-FIRST

## Abstract

Despite the involvement of subcortical brain structures in the pathogenesis of chronic pain and persistent pain as the defining symptom of knee osteoarthritis (KOA), little attention has been paid to the morphometric measurements of these subcortical nuclei in patients with KOA. The purpose of this study is to explore the potential morphological abnormalities of subcortical brain structures in patients with KOA as compared to the healthy control subjects by using high-resolution MRI. Structural MR data were acquired from 26 patients with KOA and 31 demographically similar healthy individuals. The MR data were analyzed by using FMRIB’s integrated registration and segmentation tool. Both volumetric analysis and surface-based shape analysis were performed to characterize the subcortical morphology. The normalized volumes of bilateral caudate nucleus were significantly smaller in the KOA group than in the control group (*P* = 0.004). There was also a trend toward smaller volume of the hippocampus in KOA as compared to the control group (*P* = 0.027). Detailed surface analyses further localized these differences with a greater involvement of the left hemisphere (*P* < 0.05, corrected) for the caudate nucleus. Hemispheric asymmetry (right larger than left) of the caudate nucleus was found in both KOA and control groups. Besides, no significant correlation was found between the structural data and pain intensities. Our results indicated that patients with KOA had statistically significant smaller normalized volumes of bilateral caudate nucleus and a trend toward smaller volume of the hippocampus as compared to the control subjects. Further investigations are necessary to characterize the role of caudate nucleus in the course of chronicity of pain associated with KOA.

## Introduction

Knee osteoarthritis (KOA) is a prevalent disabling condition in adults throughout the world, resulting in functional limitations, disability, reduced quality of life, and substantial healthcare costs (Felson et al., 1987; Cooper et al., 2013; Bannuru et al., 2015; Riddle et al., 2015; Sharma et al., 2015). Persistent pain is the defining symptom of KOA (Neogi et al., 2009; Nguyen et al., 2011; Malfait and Schnitzer, 2013), which increases with aging, higher levels of helplessness, and lower levels of education or income status. Experience of pain in KOA is associated with the presence of radiographic changes and with increased mortality, morbidity, and functional dependence on others. Recent advances in neuroimaging and neurophysiology provide new insights into the pathogenesis of chronic pain (Schmidt-Wilcke, 2015). Previous morphometric studies in patients with chronic pain suggest that pain may be associated with structural changes in the cortical and subcortical brain regions in many chronic pain syndromes with different pain locations and etiologies (Luchtmann et al., 2014; Mao et al., 2014; Reschetniak et al., 2014; Mao and Yang, 2015). Although chronic pain is a hallmark of KOA, relatively little is known about its properties and representation in the brain.

The epidemiology of the osteoarthritis (OA) is complex and multifactorial with genetic, biological, and biomechanical components (Glyn-Jones et al., 2015). The incidence of OA is rising because of the aging population and the epidemic of obesity (Bijlsma et al., 2011). Conservative treatment approaches include analgesic drugs, physical therapy, intra-articular injection of steroids, and viscosupplementation (Bellini and Barbieri, 2015). Among the pharmacological therapy for KOA, oral non-steroidal anti-inflammatory drugs (NSAIDs) act rapidly and are recommended for the management of KOA. However, NSAIDs are frequently associated with serious side effects, such as bleeding and gastrointestinal ulcers (Ishijima et al., 2014). Non-surgical interventions are not sufficient to control chronic severe KOA pain, either. Despite efforts over the past decades to develop markers of disease, still-imaging procedures and biochemical marker analyses need to be improved and possibly extended with more specific and sensitive methods to reliably describe disease processes, and to follow the course of disease and treatment effectiveness (Bijlsma et al., 2011).

Structural and functional MRI provides tools for understanding the mechanism underlying the brain-pain associations. Evidence from preclinical and clinical data supports a role for subcortical brain structures on chronic pain processing (Chudler and Dong, 1995; Borsook et al., 2010; Mao and Yang, 2015). The subcortical structures are composed of several nuclei involved in the execution of motor, cognitive and emotional processing (Alexander and Crutcher, 1990). As a limbic brain area, for example, the amygdala has emerged as an important brain center for the emotional affective dimension of pain and pain modulation (Neugebauer, 2015). The thalamus volume is suggested to be reversible in the painful hip OA after arthroplasty (Gwilym et al., 2010). Brain glial activation including thalamus was seen in chronic pain patients (Loggia et al., 2015). However, the morphological changes of subcortical structures – pivotal contributors to the pain matrix (Barker, 1988; Borsook et al., 2010) – have not been thoroughly described in patients with KOA.

Only a few studies have examined the brain responses in patients with knee or hip OA. Evidence from neuroimaging has indicated the involvement of subcortical structures in OA pain (Gwilym et al., 2010; Parks et al., 2011; Gimenez et al., 2014). For example, Baliki et al. (2008) suggested that brain activities in not only cortical (secondary somatosensory, insular and cingulate cortices) but also subcortical (thalamus, putamen, and amygdala) areas are associated with painful KOA, which are distinct from brain activities in chronic low back pain. Baliki et al. also found that lidocaine patch treatment modulates distinct brain circuitry in each of the two pain conditions. Another study showed distinct brain activity involved in evoked and spontaneous pain, and the latter engaged prefrontal-limbic regions corresponding to brain areas observed in other chronic pain conditions (Parks et al., 2011). Abnormal organization of the motor cortex has been reported in patients with KOA (Shanahan et al., 2015). Moreover, Wartolowska et al. (2012) demonstrated a larger volume of the caudate nucleus and nucleus accumbens in patients with rheumatoid arthritis (RA) as compared to healthy controls by automated segmentation tools. However, OA is a degenerative joint disease or wear-and-tear arthritis caused by the breakdown of joint cartilage, while RA is a chronic, autoimmune arthritis. Different pathogenesis might cause distinct brain responses. It remains unclear whether the subcortical morphology in KOA is different from those observed in RA. In addition, measurements of the surface morphology of the subcortical structures with automated segmentation tool are scarce in patients with KOA.

Based on the previous studies, as chronic pain is a major symptom linking to neuroplasticity associated with KOA, we hypothesized that potential abnormal subcortical morphology might exist in KOA. An assessment of subcortical brain morphology may provide an objective means helpful to evaluate the mechanism underlying the pathogenesis of an OA state. We aimed to examine the morphological abnormalities of subcortical structures in patients with KOA. Both volumetric analysis and surface analysis were performed to characterize the morphological differences between KOA and control groups. Relationship between MR data and the pain characteristics was measured.

## Materials and Methods

### Subjects

Twenty-six individuals with KOA (22 females; mean ± SD: 55.5 ± 9.1 years) and 31 demographically similar healthy subjects (26 females; mean ± SD: 53.1 ± 6.4 years) were included in the study. All patients were recruited from the Outpatient Clinic of Pain in our hospital. All of the patients were included if they fulfilled the criteria of the American College of Rheumatology for the classification of OA of the knee (Altman et al., 1986) and had no history of other pain conditions. The duration of knee pain was longer than 6 months with a pain magnitude of at least 3/10 on a visual analog scale (VAS). The 31 healthy volunteers were recruited via advertisements. All subjects were right-handed. Written informed consents were obtained from all participants. This study was approved by the Research Ethics Committee of the University. Participants were excluded if they had concomitant neurological, psychiatric disorders, such as hypertension, diabetes, coronary disease, or MR contraindications. No participants used antidepressants.

The KOA pain characteristics were assessed using the short form of McGill Pain Questionnaire (SF-MPQ) (Melzack, 1987), in which subjects rated the intensity of pain on a VAS from 0 to 10 (0 = no pain and 10 = maximum imaginable pain) on the day of the scan. The Hamilton depression (HAMD) scale (Endicott et al., 1981) was used to evaluate the affective state in all groups.

### Image Acquisition

We performed MRI brain scans on a 3-T GE MR scanner (GE Signa HDX, Milwaukee, WI, USA) equipped with an eight-channel head coil. The anatomic T1-weighted images were acquired using a 3D T1-weighted fast spoiled gradient echo (FSPGR) sequence with the following parameters: repetition time (TR) = 10.8 ms, echo time (TE) = 4.8 ms, matrix size = 256 × 256, FOV = 256 mm × 256 mm × 140 mm, slice thickness = 1 mm, space between slices = 0; 140 axial slices, and voxel size = 1 mm × 1 mm × 1 mm. MR imaging was performed while the participants were not experiencing pain.

### Data Processing

Data analysis was performed by using FSL tools (FMRIB Software Library^1^) (Jenkinson et al., 2012). First, SIENAX (part of FSL 5.0) (Smith et al., 2002, 2004) was used to obtain the volumes of neocortical gray matter (GM), total GM, white matter (WM), cerebral spinal fluid (CSF), total intracranial volume (TIV), and a volumetric scaling factor. Second, the absolute tissue volumes of the subcortical structures (including the caudate nucleus, putamen, pallidum, hippocampus, amygdala, thalamus, and nucleus accumbens) were estimated from the T1-weighted 3D FSPGR by using FMRIB’s integrated registration and segmentation tool (FIRST) (part of FSL 5.0, FMRIB Software Library^2^) (Patenaude et al., 2011). At last, normalized volumes of subcortical structures were thus obtained by multiplying those estimated volumes from FIRST by the volume scaling factor from SIENAX. Only the normalized volumes were involved in the subsequent statistical analysis.

Moreover, we performed surface analyses to characterize the portions of each structure that contributed most to the observed differences in global volume analysis across the two groups. The latest version of FIRST (in FSL 5.0.0) provides a new way to perform vertex analysis. The vertex-wise *F* statistics were calculated in the new method (Patenaude et al., 2011). Besides, there is a traditional surface-based vertex analysis method that was included in FSL, and the results from traditional surface-based vertex analysis contain vectors are able to display the direction of the group difference. We performed both vertex analyses and used appropriate correction methods for the multiple comparisons in the two methods. The cluster-based multiple comparison correction after controlling for family-wise error rate (FWE) was used in the new vertex analysis; and false discovery rate (FDR) correction was used in the traditional vertex analysis. In both vertex analyses, age and gender were as covariates.

### Statistical Analysis

Statistical analysis was performed with the Statistical Package for the Social Sciences for Windows (version 13.0; SPSS, Chicago, IL, USA). Differences of demographic and psychometric variables between the two groups were tested by the χ^2^ test, Student’s *t*-test, and Mann–Whitney *U* test accordingly depending on the homogeneity of variance and normality of the data. Data from SIENAX were analyzed through analysis of covariance (ANCOVA), with age and gender as covariates. Some pictures were drawn in OriginPro 8 software (OriginLab Corporation, Northampton, MA, USA).

### Volumetric Analysis for Subcortical Structures

In order to decrease the number of comparisons and due to the high correlations between the volumes of the left and right subcortical structures (Pearson coefficient, *r* values ranging from 0.464 for the nucleus accumbens to 0.944 for the thalamus), we added the volumes of the left and right sides for each structure and yielded seven data for one individual. After that, each individual’s volume measurements were standardized according to the mean value of control group using a *Z*-score transformation. Then, a repeated measures analysis of variance (ANOVA) was performed to estimate group differences with a within-group factor (structure: caudate nucleus, putamen, pallidum, hippocampus, amygdala, thalamus, and nucleus accumbens) and one between-group factor (group: control vs. KOA). *Post hoc pairwise* comparisons were carried out with *Bonferroni* corrections (for structure). If only the group differences were significant, further analysis was carried out to characterize which structure contributes most to the group difference. Hemispheric asymmetry of the subcortical structure showing significant differences was evaluated by paired *t* test for the normalized volumes of the two hemispheres in both groups. For all non-FSL analyses, a significance level of *P* < 0.05 was considered statistically significant. Age and gender were as covariates in the repeated measures ANOVA.

In addition, correlations between volume data, surface data, and pain characteristics were analyzed after adjusting the effect of age and gender.

## Results

### Demographic, Clinical, and MR Imaging Characteristics

A total of 26 patients with KOA and 31 healthy controls were enrolled in this study. Patients with KOA did not differ significantly as for age and gender. For data from SIENAX, the TIV in KOA group was significantly smaller than in the control group (*P* < 0.05). No other significant between-group differences were found as for the volumes of the neocortical GM, total GM, WM, and CSF. The information was summarized in Table 1.

**Table 1:** **Summary of the demographic and clinical data**.

Item	Group		*P*
	Controls	KOA	
Number of subjects	31	26	
Number of females (%)	26 (83.9%)	22 (84.6%)	1^b^
Age (years)	53.1 ± 6.4^a^	55.5 ± 9.1	0.25
Age range (years)	40–64	39–68	
HAMD score	3.6 ± 2.8	4.7 ± 2.2	0.13
VAS score		4.5 ± 1.8	
SF-MPQ score		13.2 ± 5.2	
Pain duration (years)		7.3 ± 9.3	
Neocortical gray matter volume	571.9 ± 22.8	559.5 ± 31.3	0.18
Total gray matter volume	717.6 ± 51.5	693.9 ± 46.6	0.06
White matter volume	673.8 ± 25.4	665.4 ± 36.8	0.46
Total intracranial volume	1430.9 ± 38.9	1398.7 ± 57.3	0.03

*VAS, visual analog scale; HAMD, Hamilton depression; SF-MPQ, short-form McGill Pain Questionnaire; KOA, knee osteoarthritis*.

*The between-group *P* values for age and HAMD scores were obtained using independent samples *t* test. For data from SIENAX, *P* values were calculated using analysis of covariance with age and gender as covariates*.

*^a^Mean ± SD. The volume is reported in cubic centimeters*.

*^b^Assessed using the χ^2^ test, two-sided*.

### Results from Volumetric Analysis

**Figure 1:**
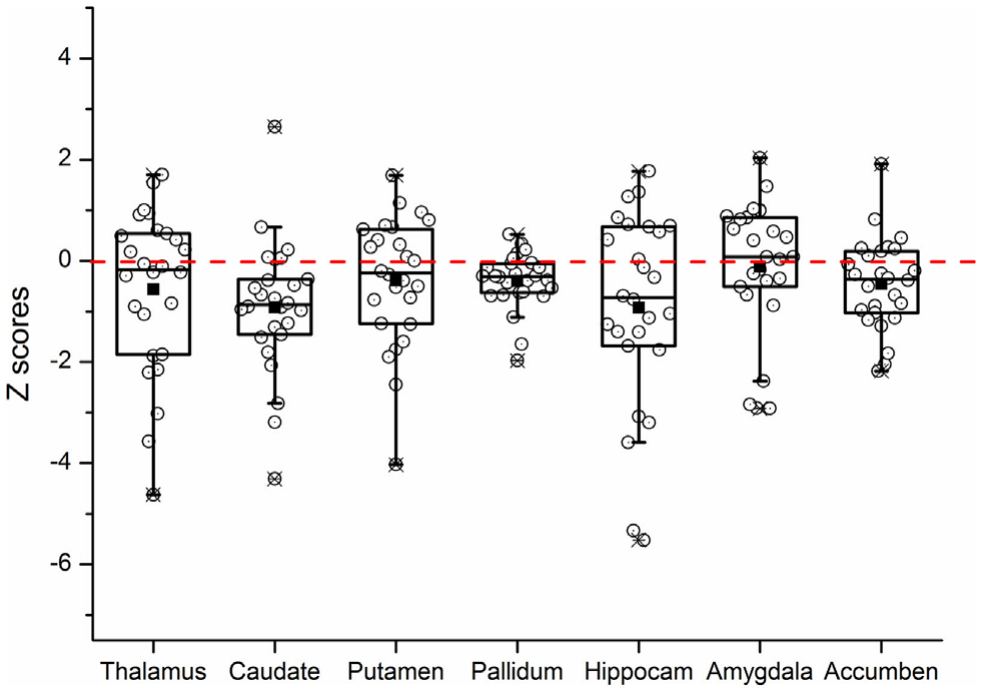
**Box-and-whisker plot showing the distribution of the *Z* scores of all subcortical brain structures in KOA group**. The upper and lower edges of the box (the hinges) mark the 25th and 75th percentiles (i.e., the central 50% of the values fall within the box), the distance between these hinges being referred to as the H spread, and the “whiskers” extend from the box and show the range of values that fall within 1.5 H spreads. The open circles represent *Z* scores of individual patients. The filled squares indicate the group means. The value 0 and the red dashed line represent the mean volume of normal controls. “Hippocam” represents “hippocampus.”

**Figure 2:**
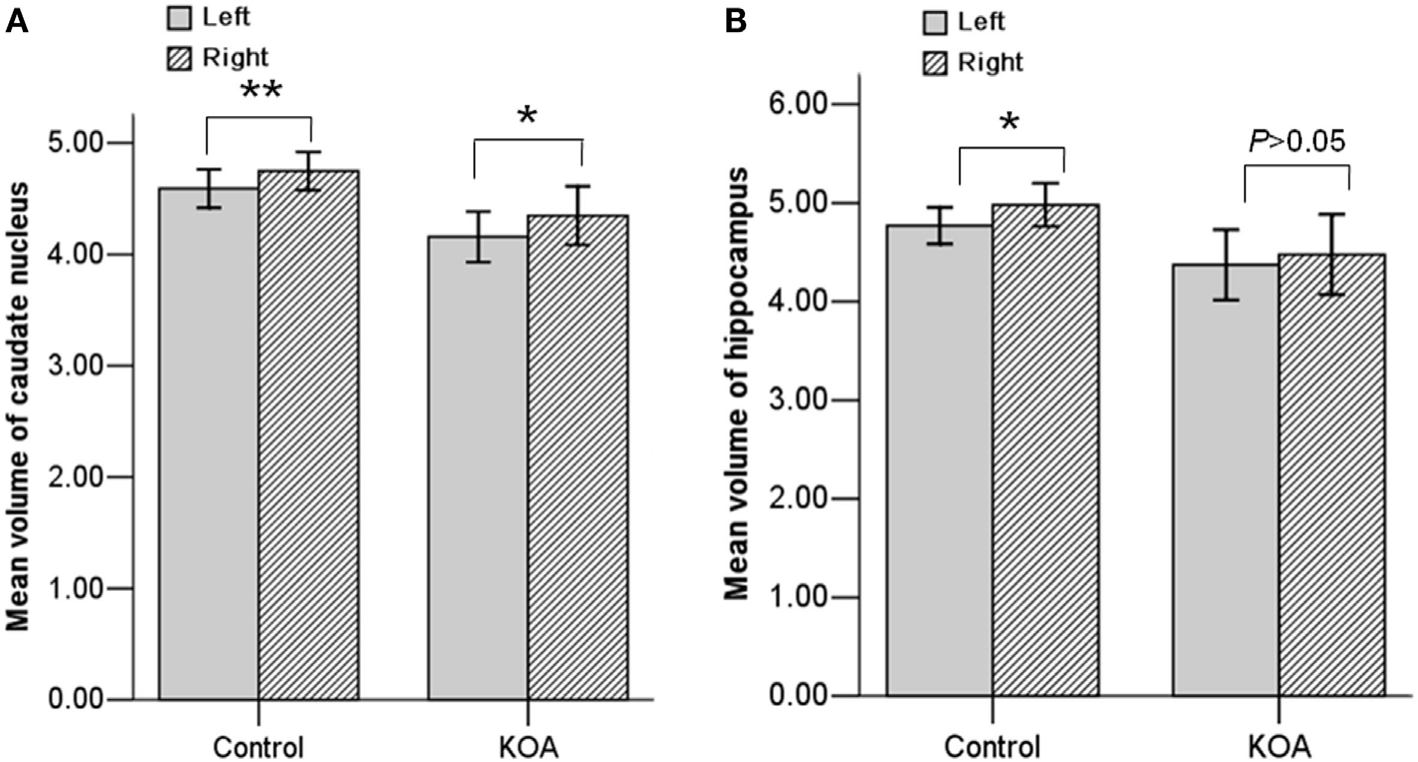
**Bar graph showing the mean volumes of the caudate nucleus (A) and hippocampus (B) at each hemisphere in both KOA and control groups**. **P < 0.01; *P < 0.05. Error bars: ±2.00 SE.

Repeated measures ANOVA for *Z* scores of the subcortical structures yielded a significant between-group difference [*F*(1, 53) = 5.273, *P* = 0.026]. The group × structure interaction was not significant (*P* = 0.177). A box-and-whisker plot was shown in Figure 1. The results indicated that the *Z* scores of the volume data for caudate nucleus and hippocampus were smaller among all subcortical structures in patients with KOA than in healthy controls. Therefore, further ANCOVA was performed with the normalized volumes of the caudate nucleus and hippocampus, after controlling the effects of age and gender. The results indicated that patients with KOA had significant smaller volumes of caudate nucleus [*F*(1, 53) = 8.93, *P* = 0.004] and hippocampus [*F*(1, 53) = 5.16, *P* = 0.027] than in healthy controls. The significant level was set at *P* < 0.025 due to the two comparisons. Therefore, only the results of caudate nucleus were reported and the MR data of it was enrolled into the subsequent analysis. Moreover, paired *t* test revealed a significant hemispheric asymmetry for the caudate nucleus in KOA (*t* = −2.478, *P* = 0.011) and controls (*t* = −2.867, *P* = 0.008) (Figure 2A). The hemispheric asymmetry of hippocampus was significant in the healthy controls (*t* = −2.058, *P* = 0.048), while this trend was not evident in KOA (*t* = −1.228, *P* = 0.231) (Figure 2B). The volumes of the subcortical structures are shown in Table 2.

**Table 2:** **Normalized volume of the caudate nucleus and hippocampus**.

Side	Structure	Group	
		Controls	KOA
Left	Thalamus	10.38 ± 0.86^a^	9.91 ± 1.28
	Caudate nucleus	4.59 ± 0.48	4.16 ± 0.58
	Hippocampus	4.77 ± 0.52	4.37 ± 0.90
	Amygdala	1.52 ± 0.29	1.47 ± 0.40
	Putamen	6.80 ± 0.74	6.63 ± 0.96
	Nucleus accumben	0.65 ± 0.14	0.59 ± 0.13
	Pallidum	2.54 ± 0.58	2.31 ± 0.31
Right	Thalamus	10.18 ± 0.76	9.76 ± 1.27
	Caudate nucleus	4.75 ± 0.48	4.35 ± 0.67
	Hippocampus	4.98 ± 0.61	4.48 ± 1.04
	Amygdala	1.43 ± 0.33	1.42 ± 0.42
	Putamen	6.60 ± 0.72	6.22 ± 0.91
	Nucleus accumben	0.53 ± 0.11	0.49 ± 0.12
	Pallidum	2.50 ± 0.54	2.30 ± 0.31
Bilateral sum	Thalamus	20.56 ± 1.59	19.67 ± 2.54
	Caudate nucleus	9.34 ± 0.91	8.51 ± 1.2
	Hippocampus	9.75 ± 0.98	8.85 ± 1.89
	Amygdala	2.95 ± 0.59	2.88 ± 0.79
	Putamen	13.4 ± 1.42	12.86 ± 1.8
	Nucleus accumben	1.18 ± 0.22	1.08 ± 0.2
	Pallidum	5.05 ± 1.11	4.61 ± 0.61

*KOA, knee osteoarthritis*.

*^a^Mean ± SD, the volume is reported in cubic centimeters*.

### Results from New Vertex-Based Shape Analysis

**Figure 3:**
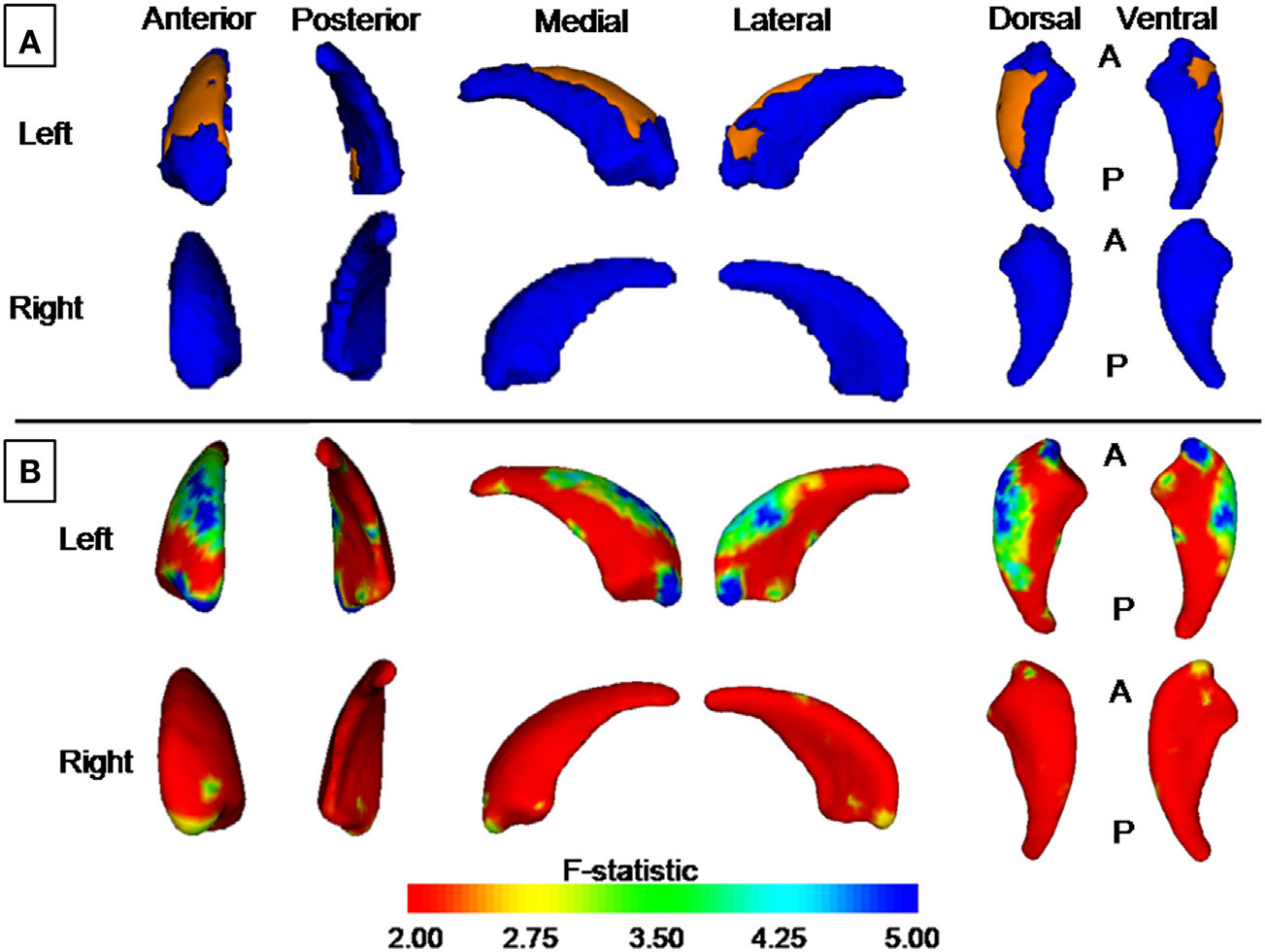
**Group differences revealed by vertex-based surface analyses**. **(A)** Results from new vertex analysis. The regions in orange represent the part of the caudate nucleus shown to be smaller in patients with KOA than in healthy controls. **(B)** Results from traditional vertex analysis. An increase from red to blue indicates a transition from lower to higher statistical significance (uncorrected). The color bar indicates the vertex-wise *F* statistic (based on Pillai’s Trace).

The new vertex analyses revealed a significant global difference between groups in the left caudate nucleus arose mainly from shrinkage of the dorsal part of the caudate nucleus (*P* < 0.05, corrected, Figure 3A), after controlling the effect of age and gender.

### Results from Traditional Vertex-Based Shape Analysis

**Figure 4:**
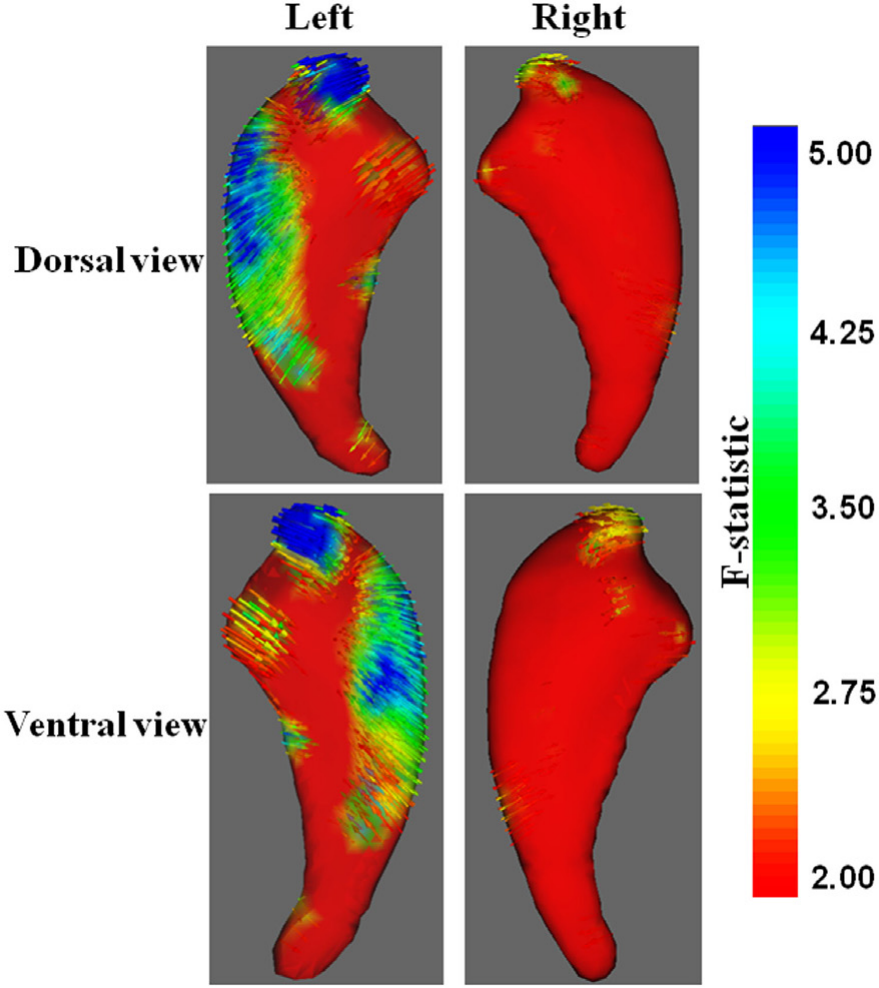
**Vector graphs of the caudate nucleus revealed by traditional vertex analysis**. An increase from red to blue indicates a transition from lower to higher statistical significance. Small arrows shown on the surface indicated the direction of the changes. Arrows pointing inward indicated that the volumes of the caudate nucleus in patients with KOA were smaller than that of the control group.

Traditional vertex analysis yielded a similar group difference as the new vertex analysis. A similar regional difference appeared at the head and dorsal part of the body of the caudate nucleus (Figures 3B and 4, uncorrected).

### Correlation Analysis

No significant correlation was observed between the volume data of the caudate nucleus and pain characteristics.

## Discussion

The main findings of the study revealed that patients with KOA had significant smaller normalized volume of bilateral caudate nucleus and a trend toward smaller volume in the hippocampus as compared to the healthy controls. These results were confirmed by volumetric analysis, and vertex-based shape analysis provided further insight into the nature of this volume difference, involving mainly the body of the caudate nucleus. Besides, hemispheric asymmetry (right larger than left) of the volumes of caudate nucleus was found in both KOA and control groups.

As one of the basic structures that make up the basal ganglia, the caudate nucleus, along with the putamen, globus pallidus, and thalamus, constitutes a system that is responsible largely for voluntary movement, learning, memory, sleep, and social behavior (Grahn et al., 2008; Hochman et al., 2011). The caudate nucleus is crucial on the evaluation of the agreement between the action and the outcome, as well as planning and performing tasks necessary to achieve complex goals. For example, Grahn et al. (2008) reported that the caudate nucleus contributed to behavior through the excitation of correct action schemas and the selection of appropriate subgoals based on an evaluation of action outcomes. Both processes are fundamental to successful goal-directed action. Another study by Robinson et al. (2012) found that different parts of the caudate nucleus were associated with distinct function: behavioral filtering resulted in cognition and emotion-related structures and networks primarily localized to the head of the caudate nucleus, while perceptual and action-specific regions localized to the body of the caudate. In KOA, the functions including perception and execution of the caudate nucleus may be disrupted by joint pain; therefore, the intended movements might be limited (Grahn et al., 2008). The smaller volume of the caudate nucleus in patients with KOA may partly be explained by the reduced movement due to chronic pain in such a disease state.

Reduced caudate nucleus volumes have been reported in several psychiatric disorders, such as attention deficit hyperactivity disorder (Castellanos et al., 1994), schizophrenia (Crespo-Facorro et al., 2007), Tourette syndrome (Bloch et al., 2005), chorea-acanthosis (Henkel et al., 2006), etc. Likewise, structural abnormality of the caudate nucleus has been reported in patients with RA (Wartolowska et al., 2012). However, our findings are different from those reported by Wartolowska et al. Increased GM in the caudate nucleus and nucleus accumbens was found in RA, but smaller volume of the caudate nucleus were seen in KOA. Different pathogenesis and different clinical characteristics of RA and KOA might contribute to the discrepancy.

Hemispheric asymmetry of basal ganglia has been repeatedly reported in previous studies (Ji and Neugebauer, 2009; Lucarelli et al., 2013; Guadalupe et al., 2014), with a rightward asymmetry of the caudate nucleus and leftward asymmetry of the putamen and globus pallidus (Wyciszkiewicz and Pawlak, 2014). Consistent with previous findings, we found a rightward hemispheric asymmetry (right larger than left) of the volumes of caudate nucleus both in KOA and control groups. By surface analysis, only the right caudate nucleus displayed significant group difference after FWE correction. We suspected that the predominance of the right caudate nucleus involved in the behavior processing might make it susceptible to OA pain.

There is a trend toward smaller volume in the hippocampus in patients with KOA as compared to healthy controls. Hippocampal volumetry is attracting more and more attention for understanding the neural basis underlying many neuropsychiatric diseases, such as mesial temporal lobe epilepsy (Pardoe et al., 2009), Alzheimer’s disease (Thomann et al., 2012), major depression (Opel et al., 2014), etc. Hippocampal volume reduction has been found to be related to persistent pain (Mutso et al., 2012) due to the increased anxiety, depression, and deficits in learning and memory. Moreover, increased regional cerebral blood flow (rCBF) has been found in hippocampus and other cortical/subcortical structures of painful patients with OA using arterial spin-labeled MRI (Howard et al., 2012). So far, no relative report was published about the structure and function measurement of hippocampus in patients with KOA. In the present study, the volume reduction of hippocampus could not withstand the stringent correction for multiple comparisons during the surface analysis. Further study combining function analysis might be helpful to characterize the hippocampal role in the pathogenesis of KOA pain.

The pathogenesis underlying the group difference in the subcortical brain morphology is unknown. KOA has complex etiologies. Inflammation is generally regarded to be prevalent in painful KOA (D’Agostino et al., 2005). Inflammation is thought to be involved in the major structural changes of the joint, causing pain and disability in patients with KOA. Several systemic markers, such as C reactive protein (CRP) (Jin et al., 2015), Interleukin-1 receptor antagonist (IL1Ra) (Attur et al., 2015), IL-6 (Beavers et al., 2015), and other serum markers play crucial role in the development and progression of OA. Inflammation can lead to visceral and musculoskeletal hyperalgesia, irrespective of biological sex (Wegner et al., 2015). Consistent with these findings, the prevalence of the neuropathic pain is reported to be over 25% in knee arthritis in Canada (Hochman et al., 2011). The neuropathic pain may be generated and/or maintained by structural changes in cortical and subcortical brain structures (Gustin et al., 2011), which were severer than that in non-neuropathic pain. The neuropathic pain components in KOA might contribute partly to the observed volume reduction of those subcortical nuclei. Future studies should be conducted to dissect the impact of neuropathic and non-neuropathic pain on brain structure. Combination of inflammatory and neuroimaging markers will eventually be beneficial to explore the pathogenesis of painful KOA.

Evidence from preclinical and clinical data suggested a potential link between inflammation and neuropsychiatric diseases (Tansey, 2010; Buga et al., 2013; Popa-Wagner et al., 2014; Sandu et al., 2015). For example, Popa-Wagner et al. suggested that inflammation may contribute to treatment resistance in depression by triggering microglial activation and subsequent neuroinflammation in the elderly (Popa-Wagner et al., 2014). Coincidentally, brain glial activation has been established to play a key role in the establishment and maintenance of persistent pain not only in preclinical persistent pain models but also in clinical chronic pain patients (Loggia et al., 2015), which may explain partially the inefficiency of anti-inflammation therapy in patients with chronic pain. Uncovering the inflammation–pain interactions may serve to guide future studies of pathophysiology and management of a variety of persistent pain conditions. Inflammation will be combined in the future study to understand the pathophysiology of the volume reduction of caudate nucleus in painful KOA.

Recent development in automated software-based segmentation now allows us to use MRI to obtain estimates of subcortical volumes without manual input (Patenaude et al., 2011). In the present study, three methods were used in this study to measure the morphology of the caudate nucleus: the volumetric analysis, new version of vertex analysis, and traditional vertex analysis. With all the three methods used, we consistently observed smaller volume of the left caudate nucleus of KOA patients. The differences were not always bilateral, which might be related to the innate asymmetry of the caudate nucleus and their increasing with age (Yamashita et al., 2011).

### Limitations

Our result should be interpreted in consideration of several limitations. First, the sample size is relatively small, which limits complex statistical analyses. Second, the cross-sectional design of the study hinders analysis of sequential causal relationships between subcortical volumes and chronic pain, and vice versa. Finally, we cannot entirely exclude the possibility that medications or comorbid affective and anxiety disorders contributed to our findings, although we did not detect any evidence for these effects. Future studies using a longitudinal design and larger samples could resolve these issues efficiently.

## Conclusion

Our results indicated that patients with KOA had statistically significant smaller normalized volumes of bilateral caudate nucleus and a trend toward smaller volume in the hippocampus as compared to the control subjects. Further investigations are necessary to characterize the role of caudate nucleus in the course of chronicity of pain associated with KOA. Continuing to broaden our understanding of this complex disease will be eventually helpful to the therapeutic advancements and prevent further disease progression.

## Author Contributions

Study design: MCP and BZL; data collection: MCP, ZXN, BZL, and ZQJ; analysis and interpretation of data: MCP and BZL; writing of the manuscript: MCP.

## Conflict of Interest Statement

The authors declare that the research was conducted in the absence of any commercial or financial relationships that could be construed as a potential conflict of interest.

## Footnotes

^1^http://www.fmrib.ox.ac.uk/fsl/

^2^http://fsl.fmrib.ox.ac.uk/fsl/fslwiki/FIRST
